# Iterative Hepatic and Pulmonary Metastasectomy in Stage IV Colorectal Cancer: Impact on Survival and Surgical Outcomes

**DOI:** 10.1245/s10434-025-18407-1

**Published:** 2025-09-30

**Authors:** Luisa Schäfer, Philipp A. Holzner, Magdalena Menzel, Gabriel J. Stöger, Andreas Gengenbach, Hans C. Hillebrecht, Francesca Reimer, Rebecca Kesselring, Uyen-Thao Le, Stefan Fichtner-Feigl, Christopher Berlin

**Affiliations:** 1 Department of General and Visceral Surgery, Faculty of Medicine, Medical Center – University of Freiburg, University of Freiburg, Freiburg, Germany; 2 German Cancer Consortium (DKTK) Partner Site, Freiburg, Germany; 3 Department of Thoracic Surgery, Faculty of Medicine, Medical Center – University of Freiburg, University of Freiburg, Freiburg, Germany; 4 IMMediate Advanced Clinician Scientist-Program, University of Freiburg, Freiburg, Germany

**Keywords:** Colorectal cancer, Surgery, Liver metastasis, Lung metastasis, Complication

## Abstract

**Background:**

Colorectal cancer frequently metastasizes to the liver and lungs. Despite curative-intent resections, recurrence rates are high. While initial hepatic and pulmonary metastasectomies have been well-studied, data are limited on outcomes following iterative resections for recurrent metastases in both locations.

**Methods:**

We retrospectively assessed the long-term outcome (> 10 years) of 97 patients who underwent iterative hepatic and pulmonary resections for recurrent colorectal cancer metastases.

**Results:**

Post initial hepatic metastasectomy survival rates showed no difference for synchronous and metachronous colorectal liver metastases. Multiple hepatic as well as pulmonary metastasectomies did ameliorate patient survival compared to single resections. Postoperative complications ≥ Clavien Dindo grade II after a second hepatic metastasectomy were associated with reduced overall survival, while complication profiles did not alter survival rates after pulmonal metastasectomies. Importantly, iterative liver and lung surgery did not increase subsequent postoperative complications, with median complication severity remaining at Clavien Dindo grade II-IIIa across successive resections.

**Conclusions:**

Our data suggest comparable benefits from hepatic metastasectomy regardless of metastatic sequence. Our findings demonstrate the safety and potential survival benefits of iterative hepatic and pulmonary resections for recurrent colorectal cancer metastases. These data support an aggressive surgical approach in selected patients with recurrent colorectal metastases.

**Supplementary Information:**

The online version contains supplementary material available at 10.1245/s10434-025-18407-1.

Colorectal cancer (CRC) is one of the most prevalent malignancies worldwide, ranking as the third most common cancer globally with an estimated 1.9 million new cases diagnosed in 2020.^1,2^ Colorectal cancer remains one of the leading causes of cancer-related mortality, with metastatic disease being the primary contributor to poor outcomes.^1–3^ Despite advances in screening and treatment, approximately 15–25% of CRC patients present with synchronous metastatic disease at initial diagnosis, and an additional 50–60% develop metachronous metastases during the course of their disease.^4^ The liver and lungs are the most common sites of distant metastases in CRC.^5^ Colorectal cancer liver metastases (CRLM) occur in nearly 50% of CRC patients, with 15–25% presenting with synchronous CRLM.^3,4,6–8^ Colorectal cancer lung metastases (CRLU) are less common but still significant, affecting approximately 10–15% of all CRC patients.^9^ While advances in systemic therapies have improved survival for patients with metastatic stage IV CRC, surgical resection of CRLM and CRLU has emerged as a potentially curative option for selected patients.^4,10,11^ Untreated stage IV CRC has a median overall survival (OS) of several months.^4,11^ With modern chemotherapy regimens, median OS has improved, but 5-year survival rates remain low.^3^ For patients undergoing complete resection of CRLM, 5-year survival rates range from 30 to 60%.^12–14^ Similarly, for patients undergoing pulmonary metastasectomy, 5-year survival rates of 40–68% have been reported.^15^

Despite curative-intent resection, many patients experience recurrence, often in the same organ.^16^ Following resection of CRLM, 60–70% of patients suffer a recurrence; in more than 50%, recurrence occurs within 2 years after initial resection.^3,16^ The management of patients with recurrent metastases post-initial metastasectomy poses a significant clinical challenge. Evidence shows that patients can also benefit from repeated hepatic resections for relapsing metastases.^10,17–19^ While initial hepatic and pulmonary metastasectomies have been extensively studied, there is a paucity of data examining the outcomes of patients undergoing repeat resections for recurrent metastases in both liver and lung.

This retrospective study evaluated the outcomes of patients who underwent iterative hepatic and pulmonary resections at our academic medical center for recurrent colorectal cancer metastases, elucidating potential benefits and limitations of this aggressive surgical approach.

## Methods

### Patient Cohort

Ninety-seven patients suffering from hepatic and pulmonary metastasized colorectal cancer with initial diagnosis between 1987 and 2014 were analyzed. All patients underwent treatment at Freiburg University Medical Center. Clinical and survival data were longitudinally collected at the Department of General and Visceral Surgery and the Comprehensive Cancer Center Freiburg.

### Study Design

Patients older than 18 years with colorectal carcinoma who underwent at least one hepatic as well as pulmonary metastasectomy due to synchronous or metachronous metastasis were included. With the exception of in situ carcinoma, all T-stages of the primary tumor were included. The primary tumor was surgically removed in all patients but not necessarily at the Freiburg University Medical Center. All metastasectomies, however, were performed at the Freiburg University Medical Center.

### Clinical Data Collection and Statistical Methods

The respective clinical data were obtained from internal medical records. Postoperative complications were assessed by using Clavien-Dindo classification. The IBM SPSS Statistics software for Windows (version 29.0.0.0) was used for exploratory statistical analyses. Comparative analyses were performed regarding survival, number and size of metastases, metastasis localization, number of surgical interventions, complications, and therapy-free intervals. If not otherwise stated, survival is defined as time beginning at the first metastasectomy. Comparative survival analyses were performed using the log-rank test (Cox-Mantel), with results presented as Kaplan-Meier curves. Patients at risk were calculated using GraphPad Prism software (version 10.1.2). For correlation analyses, data normality was assessed using the Shapiro-Wilk test. In cases of nonnormal distribution, group differences were examined by using the Kruskal-Wallis test. For each Kruskal-Wallis analysis, H-statistic, degrees of freedom (df), and *p* value are reported. Chi-square test of independence was employed to analyze associations between categorical variables, with the test statistic (*χ*^2^), sample size (*n*), degrees of freedom (df) and *p* value reported, respectively.

## Results

### Patients Characteristics

Ninety-seven patients with initial diagnosis of CRC between 1987 and 2014 were included. All patients underwent resection of the primary tumor and at least one hepatic as well as pulmonary metastasectomy due to synchronous or metachronous CRLM and CRLU. All therapeutic decisions were based on interdisciplinary expert consensus at our academic medical center. The overall mortality rate in the cohort was 81.4% (median survival estimate: 5.2 years; 95% confidence interval [CI] 3.8–6.7 years) (Supplemental Fig. 1a). Neither age, gender, body mass index, American Society of Anesthesiologists classification, T status, N status nor synchronous metastasis showed any influence on patient survival. When divided by tumor location, 26 (26.8%) patients suffered from colon carcinomas and 71 (73.2%) patients had tumors in the sigmoid or rectum. Primary tumor localization correlated with median OS. Colon carcinomas had significantly better survival than sigmoid or rectal carcinomas (median survival estimate: 11.2 vs. 5.1 vs. 4.5 years; log-rank (Cox-Mantel): *χ*^2^(2) = 7.975, *p* = 0.019) (Supplemental Fig. 1b). All patient characteristics are depicted in Table 1.

**Fig. 1:**
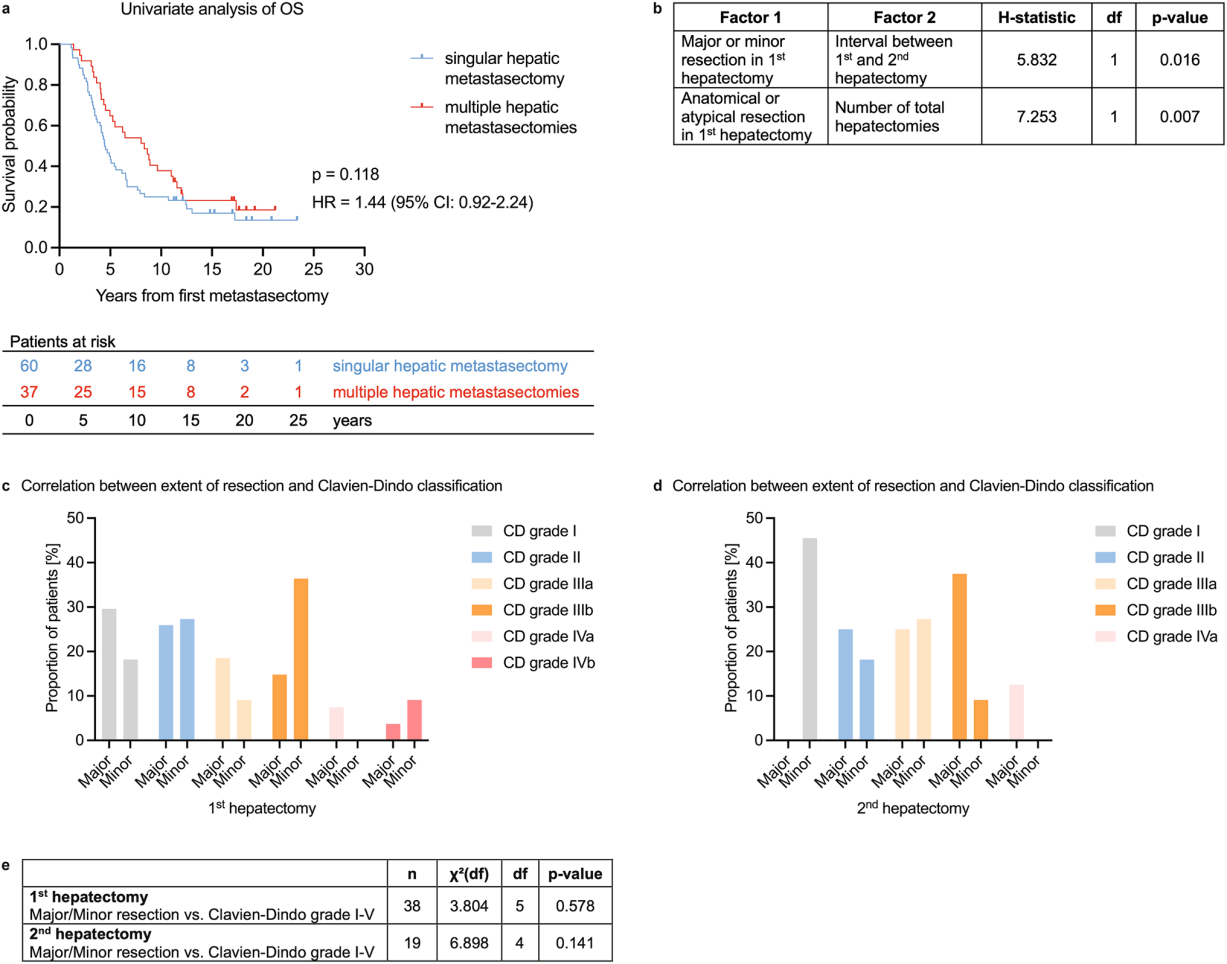
Surgical procedures liver. **a** Kaplan-Meier curve of univariate overall survival in patients with singular or multiple hepatic metastasectomies. *n* = 97, *p* = 0.118. HR (log-rank) 1.44, (95% CI 0.92–2.24). *OS* overall survival. **b** Correlation between extent of hepatic resection and relapse-free interval (interval between first and second hepatectomy) or total number of hepatic metastasectomies using Kruskal-Wallis test. df = degrees of freedom. **c**, **d** Bar chart for the correlation of the extent of resection (major vs. minor) and postoperative complications as classified by Clavien-Dindo (CD) for first (**c**) and second (**d**) hepatectomy. Major resection is defined as resection of more than three liver segments. **e** Results of chi-square test. *n* number of patients, *df* degrees of freedom, *HR* hazard ratio, *CI* confidence interval

**Table 1:** Patient demographics

	Total	Percent (%)	Range	Median survival estimate (yr)	Median survival estimate 95% CI (yr)	Log-rank (Cox-Mantel): *χ*^2^(df)	Df	Log-rank (Cox-Mantel): *p*
Patients included	97	100		5.2	3.8–6.7			
Last follow-up (September 2024)	Alive	18	18.6					
	Deceased	79	81.4					
Gender	Female	26	26.8	5.1	2.1–8.1	0.704	1	0.402
	Male	71	73.2	5.4	3.9–6.9			
Median BMI (kg/m^2^)	25.8		17.4–56.8					
BMI (kg/m^2^)	≤ 25	40	41.2	5.0	2.5–7.5	1.071	1	0.301
	> 25	57	58.8	5.2	4.0–6.4			
Median age at first diagnosis (yr)	58.3		35.4–84.4					
Age (yr)	< 60	51	52.6	5.5	3.3–7.6	0.271	1	0.603
	≥ 60	46	47.4	5.0	3.8–6.1			
ASA score	I	3	3.1	6.4	2.6–10.3	3.336	3	0.343
	II	48	49.5	5.0	2.9–7.1			
	III	45	46.4	5.2	3.2–7.2			
	IV	1	1	3.1	–			
Primary tumor localization	Colon	26	26.8	11.2	6.1–16.2	7.975	2	0.019
	Sigma	30	30.9	5.1	2.1–8.1			
	Rectum	41	42.3	4.5	3.8–5.3			
Metastases at initial diagnosis	M0	55	56.7	5.5	3.6–7.3	0.305	1	0.581
	M1	42	43.3	5.0	3.6–6.3			
	Liver	39	40.2	5.2	3.3–7.2			
	Lung	13	13.4	4.3	3.0–5.5			
	Other sites	4	4.1	2.9	1.1–4.8			
	Liver + Lung	10	10.3	4.5	2.3–6.8	3.575	2	0.167
	Only liver	26	26.8	6.2	1.7–10.6			
	Only lung	2	2.1	2.3	–			
Pathology								
Grading primary tumor	G1	1	1	3.2	–	2.515	2	0.284
	G2	81	83.5	5.2	3.8–6.7			
	G3	15	15.5	6.5	3.4–9.6			
pT at initial diagnosis	T1	2	2.1	5.4	–	0.458	3	0.928
	T2	9	9.3	7.7	0.0–16.4			
	T3	73	75.3	5.6	3.7–7.4			
	T4	13	13.4	4.1	2.3–5.9			
pN at initial diagnosis	N0	34	35.1	8.0	5.0–11.1	3.599	2	0.165
	N1	39	40.2	4.5	3.4–5.7			
	N2	24	24.7	4.3	3.2–5.3			
Resection status primary tumor	R0	95	97.9	5.4	3.9–6.9	3.335	1	0.068
	R1	2	2.1	1.4	–			
	R2	0	0	–	–			
Resection status overall *(*n* = 82)	R0	63	76.8	5.5	3.7–7.2	0.739	1	0.390
	R1	19	23.2	6.2	4.1–8.2			
	R2	0	0	–	–			

*Indication if number of patients differs from all patients included

*BMI* body mass index

### CRLM and Therapeutic Potential of Iterative Hepatic Metastasectomies

Forty-two patients suffered from synchronous (< 9 months from time of first diagnosis) and 55 patients from metachronous (> 9 months from time of first diagnosis) hepatic metastasis. The sequence of hepatic metastasis in terms of synchronous or metachronous CRLM displayed no influence on overall survival (median survival estimate: 5.2 vs. 5.0 years; log-rank (Cox-Mantel): *χ*^2^(2) = 1.743, *p* = 0.187) (Supplemental Figs. 2a, b).

**Fig. 2:**
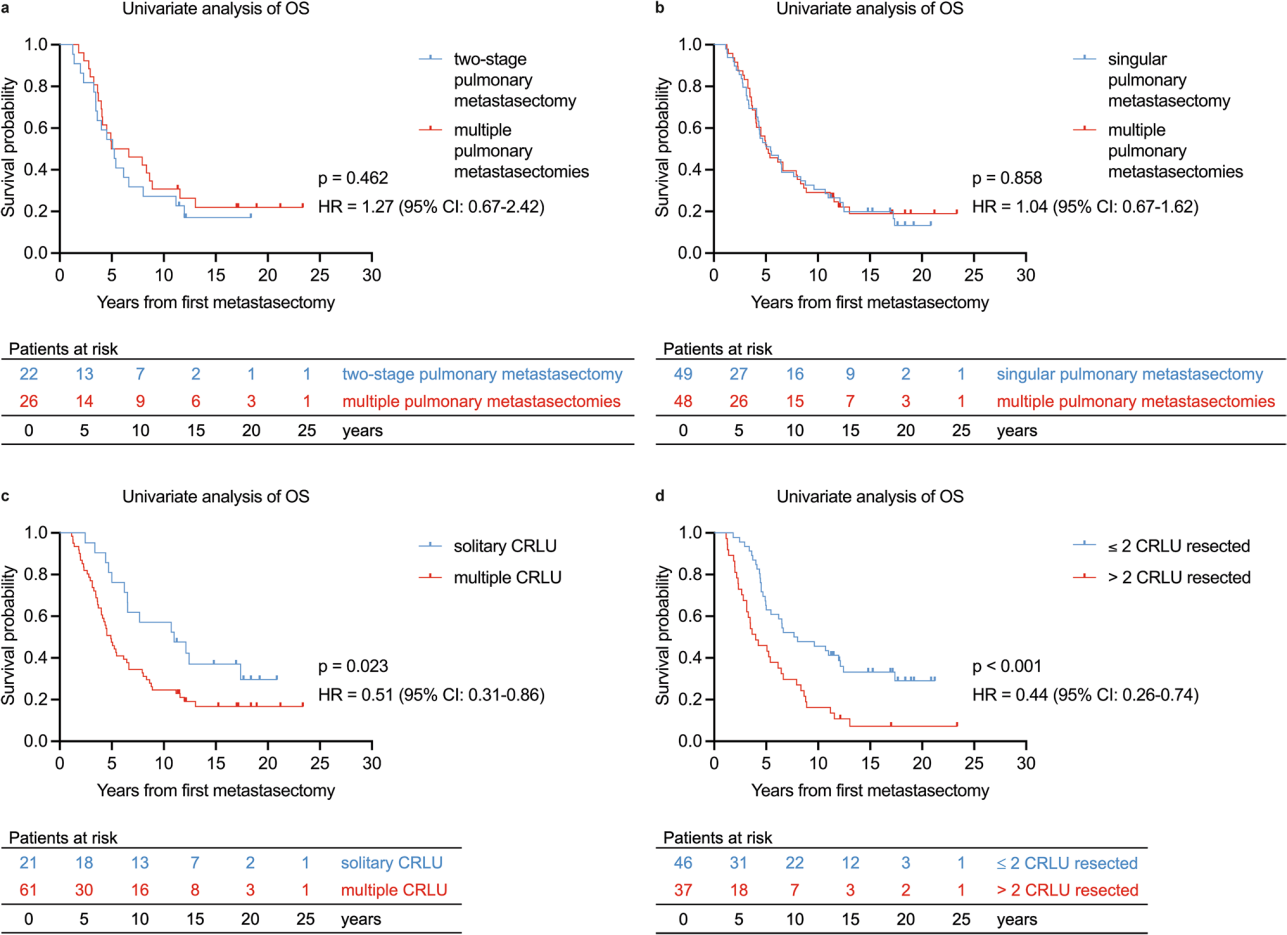
Surgical procedures lung. **a** Kaplan-Meier curve of univariate overall survival in patients with planned two-stage pulmonary metastasectomy and nonplanned repeated pulmonary resection. *n* = 48, *p* = 0.462, HR (log-rank) 1.27, (95% CI 0.67–2.42). **b** Kaplan-Meier curve of univariate overall survival in patients with singular or multiple pulmonary metastasectomies. *n* = 97, *p* = 0.858. HR (log-rank) 1.04, (95% CI 0.67–1.62). **c** Kaplan-Meier curve of univariate overall survival in patients with solitary or multiple pulmonary metastases. *n* = 82. *p* = 0.023. HR (log-rank) 0.51, (95% CI 0.31–0.86). **d** Kaplan-Meier curve of univariate overall survival in patients with resection of ≤ 2 or > 2 pulmonary metastases during first pulmonary metastasectomy. *n* = 83, *p* < 0.001, HR (log-rank) 0.44, (95% CI 0.26–0.74). *OS* overall survival, *HR* hazard ratio, *CI* confidence interval

As far as the overall hepatic tumor load is concerned, 29.3% of patients were diagnosed with a single CRLM, and 70.7% suffered from multiple hepatic lesions during the course of their disease. Among all patients, 61.9% received a single hepatic metastasectomy and 38.1% underwent multiple liver surgeries (median 1 (1–6)) (Table 3). Importantly, iterative hepatic surgery ameliorated the disparity in patient survival (median survival estimate: 4.5 (singular hepatic metastasectomy) vs. 8.4 (multiple hepatic metastasectomies) years; log-rank (Cox-Mantel): *χ*^2^(1) = 2.45, *p* = 0.118) (Fig. 1a).

A liver-first approach was performed in 3% of all cases, whereas 12.4% underwent simultaneous resection of hepatic metastases and the primary tumor. Neither size of CRLM nor intersurgical timeframes between hepatectomies during the course of disease significantly affected patient survival (Tables 2 and 3).

**Table 2:** Characteristics of CRLM, CRLU, and metastasectomies

	Total	Percent (%)	Range	Median survival estimate (yr)	Median survival estimate 95% CI (yr)	Log-rank (Cox-Mantel): *χ*^2^(df)	df	Log-rank (Cox-Mantel): *p*
Hepatic metastasis sequence (cutoff: 9 months from initial diagnosis) (survival from initial diagnosis)	Synchronous	42	43.3	5.6	3.8–7.4	0.152	1	0.696
	Metachronous	55	56.7	8.2	5.9–10.5			
Hepatic metastasis sequence (cutoff: 9 months from initial diagnosis) (survival from first metastasectomy)	Synchronous	42	43.3	5.2	3.2–7.3	1.743	1	0.187
	Metachronous	55	56.7	5.0	3.2–6.8			
Median total no. metastases *(*n* = 82)	7		2–36					
No. total metastases During total course of disease *(*n* = 82)	2	7	8.5	6.5	6.5–6.6	0.000	1	0.986
	> 2	75	91.5	5.4	4.0–6.8			
Median total number of CRLM *(*n* = 82)	2		1–13					
No. CRLM during total course of disease *(*n* = 82)	1	24	29.3	4.3	3.3–5.2	7.723	1	0.005
	> 1	58	70.7	7.9	4.8–11.0			
No. CRLM during total course of disease *(*n* = 82)	≤ 2	43	52.4	4.7	3.9–5.5	4.737	1	0.030
	> 2	39	47.6	8.4	5.2–11.5			
Median total no. CRLU *(*n* = 82)	4		1–35					
No. CRLU during total course of disease *(*n* = 82)	1	21	25.6	11.0	4.6–17.4	5.139	1	0.023
	> 1	61	74.4	4.9	4.0–5.9			
No. CRLU during total course of disease *(*n* = 82)	≤ 2	30	36.6	6.5	4.6–8.5	2.286	1	0.131
	> 2	52	63.4	4.9	3.4–6.4			
Nodal positive CRLU *(*n* = 82)	Yes	10	12.2	5.2	2.0–8.5	1.139	1	0.286
	No	72	87.8	5.5	3.9–7.1			
Median minimal diameter of CRLM [cm] *(*n* = 82)	2.5		0.7–12.0					
Median maximum diameter of CRLM (cm) *(n = 82)	3.6		0.9–17.0					
Maximum diameter of CRLM *(*n* = 82)	≤ 3.6 cm	42	51.2	6.2	4.4–7.9	0.150	1	0.698
	> 3.6 cm	40	48.8	5.2	2.4–8.0			
Median minimal diameter of CRLU [cm] *(*n* = 82)	0.8		0.0–8.0					
Median maximum diameter of CRLU (cm) *(*n* = 82)	1.5		0.3–8.0					
Maximum diameter of CRLU *(*n* = 82)	≤ 1.5 cm	43	52.4	5.2	3.4–7.1	0.050	1	0.824
	> 1.5 cm	39	47.6	6.5	2.9–10.1			
Median number of metastasectomies	3		2–8					
First metastasectomy	Liver	92	94.8	5.5	4.0–6.9	14.714	2	< 0.001
	Lung	3	3.1	4.4	1.3–7.6			
	Simultaneous	2	2.1	1.8	–			
Last metastasectomy	Liver	17	17.5	4.7	3.5–5.9	3.913	1	0.048
	Lung	80	82.5	5.6	3.8–7.3			
Median interval between primary tumor resection and first metastasectomy (mo)	11		4–101					
Interval between primary tumor resection and first metastasectomy	≤ 12 months	50	51.5	5.0	3.7–6.3	0.034	1	0.854
	> 12 months	47	48.5	6.2	4.1–8.4			
Median interval between primary tumor resection and last metastasectomy (mo)	39		1–152					
Interval between primary tumor resection and last metastasectomy	≤ 36 mo	47	48.5	3.5	3.0–4.0	21.267	1	< 0.001
	> 36 mo	50	51.5	8.6	5.4–11.9			
Interval between first hepatic and pulmonary resection	≤ 18 mo	62	63.9	4.0	3.3–4.8	10.690	1	0.001
	> 18 mo	35	36.1	9.6	6.6–12.7			
Surgical procedures for metastasectomy	hep > pul	18	18.5	8.0	4.1–12.0	0.921	2	0.631
	hep = pul	44	45.4	4.5	3.6–5.3			
	hep < pul	35	36.1	5.1	2.7–7.5			

*Indication if number of patients differs from all patients included

**Table 3:** Surgical procedures liver and postoperative complications

	Total	Percent (%)	Range		Median survival estimate (yr)	Median survival estimate 95% CI (yr)	Log-rank (Cox-Mantel): *χ*^2^ (df)	df	Log-rank (Cox-Mantel): *p*
Median no. hepatic resections	1		1–6						
No. hepatic resections	1	60	61.9		4.5	3.6–5.3	2.45	1	0.118
	> 1	37	38.1		8.4	5.3–11.4			
Liver-first approach	3	3.1		4.1	3.3–4.9	1.449	2	0.485	
Simultaneous hepatic resection with primary tumor resection	12	12.4		4.1	0.0–9.0				
Hepatic resection after primary tumor resection	82	84.5		5.2	3.7–6.8				
Two-stage hepatectomy	5	5.2		5.5	1.6–9.4				
Median interval between primary tumor resection and first hepatectomy [months] *(*n* = 82)	15		1–105						
First hepatectomy	Anatomical *(*n* = 95)	50	52.6		5.0	3.8–6.2	0.393	2	0.821
	Atypical *(*n* = 95)	30	31.6		6.2	3.9–8.5			
	Anatomical + atypical *(*n* = 95)	15	15.8		5.1	2.5–7.7			
	Major hepatic resection (> 3 segments)	52	53.6		5.2	3.1–7.4	2.517	1	0.113
	Minor hepatic resection	45	46.4		5.0	3.5–6.4			
Median no. hepatic resections following	Atypical resection in first hepatectomy	2		1–6					
	anatomical resection first hepatectomy	1		1–3					
Median interval between first and second hepatectomy (mo) *(*n* = 37)	13		1–106						
Interval between first and second hepatectomy *(*n* = 37)	≤ 13 mo	19	51.4		5.5	0.0–11.1	0.354	1	0.552
	> 13 mo	18	48.6		8.6	7.5–9.8			
Second hepatectomy *(*n* = 37)	Anatomical	18	48.7		6.4	2.0–10.9	5.452	2	0.065
	atypical	15	40.5		2.2	0.0–4.5			
	Anatomical + atypical	4	10.8		11.0	6.1–15.9			
	Major hepatic resection (> 3 segments)	13	35.1		6.2	4.5–8.0	1.392	1	0.238
	Minor hepatic resection	24	64.9		8.6	5.1–12.2			
Median interval between second and third hepatectomy (mo) *(*n* = 11)	15		2–40						
Interval between second and third hepatectomy *(*n* = 11)	≤ 15 mo	8	72.7		4.5	2.5–6.5	0.011	1	0.916
	> 15 mo	3	27.3		8.4	7.8–8.9			
Third hepatectomy *(*n* = 11)	Major hepatic resection (> 3 segments)	1	9.1		8.0	–	0.108	1	0.742
	Minor hepatic resection	10	90.9		5.5	0.0–11.4			
Clavien-Dindo first hepatectomy *(*n* = 71)	No complication	33	46.5		6.2	4.0–8.4	11.585	6	0.072
	Grade I	10	14.1		–	–			
	Grade II	10	14.1		6.2	4.2–8.1			
	Grade IIIa	6	8.5		1.9	0.0–5.3			
	Grade IIIb	8	11.3		3.3	2.6–3.9			
	Grade IVa	2	2.8		1.4	–			
	Grade IVb	2	2.8		4.5	–			
Clavien-Dindo first hepatectomy *(*n* = 71)	≤ Grade I	43	60.6		6.4	4.6–8.3	1.531	1	0.216
	> Grade I	28	39.4		4.4	3.5–5.3			
Clavien-Dindo second hepatectomy *(*n* = 31)	No complication	12	38.7		12.1	–	8.923	5	0.112
	grade I	5	16.1		8.4	0.0–19.0			
	Grade II	4	12.9		8.6	5.3–12.0			
	Grade IIIa	5	16.1		4.4	3.6–5.1			
	Grade IIIb	4	12.9		5.0	0.2–9.7			
	Grade IVa	1	3.2		3.6	–			
	Grade IVb	0	0		–	–			
Clavien-Dindo second hepatectomy *(*n* = 31)	≤ Grade I	17	54.8		12.1	6.3–18.0	5.523	1	0.019
	> Grade I	14	45.2		5.2	4.3–6.1			
Clavien-Dindo third hepatectomy *(*n* = 9)	No complication	4	44.4		8.4	–	3.321	3	0.345
	Grade I	0	0		–	–			
	Grade II	2	22.2		4.0	–			
	Grade IIIa	1	11.1		5.5	–			
	Grade IIIb	2	22.2		2.0	–			
	Grade IVa	0	0		–	–			
	Grade IVb	0	0		–	–			
Clavien-Dindo third hepatectomy *(*n* = 9)	≤ Grade I	4	44.4		8.4	–	2.517	1	0.113
	> Grade I	5	55.6		5.5	2.4–8.6			

^*^Indication if number of patients differs from all patients included.

Patients with postoperative complications after the second hepatic metastasectomy of at least CD grade II compared with patients with no or minor complications (CD grade I) showed reduced OS (median survival estimate: 12.1 vs. 5.2 years; log-rank (Cox-Mantel): *χ*^2^(1) = 5.523, *p* = 0.019). Importantly, undergoing repeated liver surgery did not result in increased severity of postoperative complications compared with a single hepatectomy. The hepatic resections resulted in complications of CD grade I-IVb, with a median severity of grade II-IIIa (first hepatectomy: CD grade I-IVb, *n* = 38; second hepatectomy: CD grade I-Iva, *n* = 19; third hepatectomy: CD grade II-IIIb, *n* = 5) (Table 3). These data reveal the therapeutic potential and safety of iterative hepatic resections for CRLM.

### Surgical Extent of Iterative Liver Surgery does not Impact Patient Survival and Safety

In our study, 53.6% of patients underwent major hepatic resection, defined as resection of more than three liver segments, during the first hepatic surgery. The extent of resection (major vs. minor) during the first hepatic intervention demonstrated no significant impact on patient survival (Table 3). Interestingly, patients who initially underwent atypical resection (31.6%) underwent a median of two repeat hepatic interventions (range 1–6) compared with those with initial anatomical resection (52.6%), who underwent a median of one repeat hepatectomy (range 1–3) (Table 3). In our cohort, a significant positive correlation was observed between major liver resection and a prolonged interval between first and second hepatic metastasectomies (H(1) = 5.832, *p* = 0.016) (Fig. 1b). Patients who underwent anatomical resections in their first procedure had significantly fewer total hepatic metastasectomies (H(1) = 7.253, *p* = 0.007) (Fig. 1b). Notably, patients undergoing major resections (35.1%) during the second hepatic metastasectomy did not suffer from significantly reduced survival compared to those with minor resections (64.9%) (median survival estimate: 6.2 vs. 8.6 years; log-rank (Cox-Mantel): *χ*^2^(1) = 1.392, *p* = 0.238) (Table 3). Furthermore, anatomical and atypical approaches in recurrent liver surgery did not influence patient survival. The extent of resection (major vs. minor) in iterative metastasectomies did not correlate with postoperative complications as classified by CD (first hepatectomy: *χ*^2^(5, n = 38) = 3.804, *p* = 0.578; second hepatectomy: *χ*^2^(4, *n* = 19) = 6.898, *p* = 0.141) (Fig. 1c, e). In our real-world surgical cohort, we observed an association between the extent of hepatic resection (major vs. minor) and the surgical approach (anatomical vs. atypical) at first metastasectomy and both time to hepatic relapse and rate of recurrence. Importantly, different types of surgical procedures do not significantly affect overall patient survival.

### Therapeutic Potential of Iterative Pulmonary Metastasectomies

Of the total patients, 50.5% underwent a single pulmonary metastasectomy (median 1 (1–7)), and 49.5% were treated by multiple resections of CRLU. Patients with bilateral CRLU predominantly underwent planned staged bilateral pulmonary resection (*n* = 22 (22.7%)). This surgical procedure did not show any significant impact on overall survival compared with patients undergoing nonplanned repeat pulmonary resections (median survival estimate: 5.1 vs. 5.0 years; log-rank (Cox-Mantel): *χ*^2^(1) = 0.542, *p* = 0.462) (Table 4; Fig. 2a). Importantly, iterative pulmonary resections were not associated with reduced long-term survival compared with single lung resections (median survival estimate: 5.1 vs. 5.5 years; log-rank (Cox-Mantel): *χ*^2^(1) = 0.032, *p* = 0.857) (Fig. 2b). Postoperative complications, documented by CD, for the first and second surgical pulmonary intervention, exhibited no impact on patient’s long-term survival (Table 4). These data indicate that iterative pulmonary metastasectomy equalizes OS for recurrent CRLU.

**Table 4:** Surgical procedures lung and postoperative complications

	Total	Percent (%)	Range	Median survival estimate (yr)	Median survival estimate 95% CI (yr)	Log-rank (Cox-Mantel): *χ*^2^ (df)	df	Log-rank (Cox-Mantel): *p*
Median no. lung resections	1		1–7					
No. pulmonary resections (survival from initial diagnosis)	1	49	50.5	8.2	4.8–11.7	0.000	1	0.984
	> 1	48	49.5	6.8	5.4–8.3			
No. pulmonary resections (survival from first metastasectomy)	1	49	50.5	5.5	3.0–7.9	0.032	1	0.857
	> 1	48	49.5	5.1	3.2–6.9			
No. pulmonary resections (survival from initial diagnosis)	≤2	79	81.4	6.4	5.3–7.5	3.843	1	0.050
	> 2	18	18.6	12.2	11.2–13.3			
No. pulmonary resections (survival from first metastasectomy)	≤2	79	81.4	4.7	4.0–5.4	4.175	1	0.041
	> 2	18	18.6	11.2	6.3–16.0			
Multiple pulmonary resections *(*n* = 48)	Two-stage bilateral pulmonary resection	22	45.8	5.1	3.5–6.7	0.542	1	0.462
	Nonplanned repeat pulmonary resection	26	54.2	5.0	0.7–9.2			
First pulmonary metastasectomy—median no. resected metastases	2		1–22					
First pulmonary metastasectomy—no. resected metastases *(*n* = 83)	1	34	41	9.6	3.4–15.8	6.984	1	0.008
	> 1	49	59	4.5	3.4–5.7			
First pulmonary metastasectomy—number of resected metastases *(*n* = 83)	≤ 2	46	55.4	7.7	3.0–12.3	11.761	1	< 0.001
	> 2	37	44.6	4.0	2.1–5.9			
First pulmonary metastasectomy—localization *(*n* = 83)	Left	34	41	6.2	4.1–8.2	0.370	2	0.831
	Right	41	49.4	5.2	3.3–7.2			
	Bilateral	8	9.6	8.8	2.8–14.9			
First pulmonary metastasectomy—LAD *(*n* = 82)	Yes	57	69.5	6.2	4.7–7.7	1.396	1	0.237
	No	25	30.5	4.5	2.8–6.3			
Median interval between first and second pulmonary metastasectomy (mo) *(*n* = 48)	4		0–54					
Second pulmonary metastasectomy—median number of resected metastases *(*n* = 45)	2		1–25					
Second pulmonary metastasectomy—no. resected metastases *(*n* = 45)	1	19	42.2	8.4	3.3–13.4	1.091	1	0.296
	> 1	26	57.8	4.5	2.8–6.3			
Second pulmonary metastasectomy—localization *(n = 45)	Left	23	51.1	5.2	1.9–8.5	1.181	2	0.554
	Right	21	46.7	6.2	3.8–8.5			
	Bilateral	1	2.2	4.5	–			
Second pulmonary metastasectomy—LAD *(*n* = 45)	Yes	36	80	5.0	3.9–6.0	1.535	1	0.215
	No	9	20	8.6	7.8–9.5			
Median interval between second and third pulmonary metastasectomy (mo) *(*n* = 17)	6		1–49					
Third pulmonary metastasectomy—median number of resected metastases *(n = 17)	1		1–6					
Third pulmonary metastasectomy—number of resected metastases *(*n* = 17)	1	9	52.9	11.6	8.2–14.9	0.006	1	0.939
	> 1	8	47.1	6.6	0.0–14.7			
Third pulmonary metastasectomy—localization *(*n* = 17)	Left	8	47.1	11.6	0.0–26.1	0.711	1	0.399
	Right	9	52.9	11.2	4.5–17.8			
	Bilateral	0	0	–	–			
Third pulmonary metastasectomy—LAD *(*n* = 17)	Yes	6	35.3	6.6	0.0–14.3	0.134	1	0.714
	No	11	64.7	11.6	7.5–15.6			
Median interval between third and fourth pulmonary metastasectomy (mo) *(*n* = 7)	12		0–45					
Clavien-Dindo first pulmonary metastasectomy *(*n* = 75)	No complication	49	65.3			9.918	5	0.078
	Grade I	9	12					
	Grade II	12	16					
	Grade IIIa	1	1.3					
	Grade IIIb	3	4.0					
	Grade IVa	1	1.3					
	Grade IVb	0	0					
	Grade V	0	0					
Clavien-Dindo second pulmonary metastasectomy *(*n* = 45)	No complication	32	71.1			2.34	4	0.674
	Grade I	5	11.1					
	Grade II	5	11.1					
	Grade IIIa	2	4.4					
	Grade IIIb	0	0					
	Grade IVa	0	0					
	Grade IVb	1	2.2					
	Grade V	0	0					
Clavien-Dindo third pulmonary metastasectomy *(*n* = 16)	No complication	10	62.5			11.878	5	0.036
	Grade I	2	12.5					
	Grade II	1	6.3					
	Grade IIIa	1	6.3					
	Grade IIIb	0	0					
	Grade IVa	1	6.3					
	Grade IVb	0	0					
	Grade V	1	6.3					

*Indication if number of patients differs from all patients included.

### Number of CRLU Impact Patient Survival

Of the total patients, 25.6% suffered from solitary and 74.4% from multiple CRLU (Table 2). A solitary CRLU during the total course of the disease was associated with improved patient survival compared with patients with multiple lesions (median survival estimate: 11.0 vs. 4.9 years; log-rank (Cox-Mantel): *χ*^2^(1) = 5.139, *p* = 0.023) (Fig. 2c).

As far as the size of pulmonary lesions is concerned, the median maximum diameter of respective metastases was 1.5 cm (range 0.3–8.0 cm). Pulmonary metastasis size demonstrated no significant impact on patient survival. In conclusion, patients with solitary CRLU demonstrate significantly improved survival outcomes compared with those with multiple lesions.

### Number of Metastases Resected in First Lung Surgery Affects Patient Survival

Of the total patients, 41% of patients had a single metastasis resected during the first pulmonary metastasectomy, whereas 14.5% of patients underwent resection of two CRLU. Resection of a maximum of two metastases during the first pulmonary metastasectomy was associated with significantly improved OS compared with resection of multiple metastases (median 2 (1–22)) (median survival estimate 7.7 vs. 4.0 years; log-rank (Cox-Mantel): *χ*^2^(1) = 11.761, *p* < 0.001) (Table 4; Fig. 2d). These data suggest that patients with initially limited pulmonary metastasis show improved overall survival.

## Discussion

The management of metastatic CRC has evolved considerably during recent years, with surgical interventions playing an important role. Synchronous CRLM occur in approximately one third of patients with CRC.^20^ Surgical resection of CRLM is crucial for long-term survival.^20^ Reported 5-year survival rates for patients following hepatic and pulmonary vary between 39 and 50%.^21–23^ Our patient cohort demonstrated comparatively good outcomes, with a median overall survival of 5.2 years and a 5-year survival rate of 51.5%. This exceptional result is most likely attributable to rigorous patient selection and strict adherence to surgical indications based on interdisciplinary consensus.

Hand in hand with already published data, our cohort of patients with both hepatic and pulmonary metastases similarly demonstrated markedly superior survival in cases with colon primaries (median survival: 11.2 years) compared with sigmoid (5.1 years) and rectal (4.5 years) carcinomas.^24^ In contrast to some previously published studies, this finding indicates a different survival pattern between right- and left-sided colon carcinomas, even after adjusting for tumor location. Several studies have shown improved outcomes for left-sided CRC, including rectal cancers, relative to right-sided tumors.^25–27^ Only a few studies report worse survival for rectal carcinoma; for instance, Duraes et al. observed improved survival rates for right-sided colon cancer compared with rectal cancer after adjusting for age, gender, American Society of Anesthesiologists score, chemotherapy, and pathological stage.^28^ Paschke et al. proposed that colon and rectal cancers may be biologically distinct entities; however, they concluded that the prognosis of rectal cancer is at least equivalent to that of colon cancer.^29^

Our findings suggest that in patients with dual-site metastases undergoing repeated surgical interventions, a relatively selective group that is underrepresented in current literature, rectal cancer may be associated with poorer outcomes. This highlights the potential prognostic significance of the primary tumor location in advanced metastatic CRC.

Our analysis reveals that patients suffering from synchronous CRLM may benefit similarly to patients with metachronous CRLM from hepatic metastasectomy. This finding challenges the traditional view that synchronous CRLM are more aggressive and associated with shorter survival compared to metachronous metastases. The original approach for patients with synchronous CRLM, which involves resection of the primary tumor followed by systemic therapy and subsequent hepatic metastasectomy when feasible, is meanwhile frequently abandoned.^20^ Current management often involves either simultaneous resection of metastases and primary tumor or a liver-first approach, frequently preceded by neoadjuvant systemic therapy to potentially downsize metastases and improve resectability.^20,30–32^ The liver-first approach is particularly used for high hepatic tumor burden with multiple, bilobar metastases.^33^ Brouquet et al. conducted a comparative analysis of these three strategies for synchronous CRLM management, reporting comparable mortality, morbidity, and survival rates.^32^ Interestingly, their study identified tumor size exceeding 3 cm as a prognostic factor associated with survival, a finding not corroborated in our cohort.^32^ In the past, synchronous CRLM have been associated with poorer prognosis compared to metachronous disease.^23,34,35^ However, recent studies have challenged this notion. Wisneski et al. reported significantly improved survival in contemporary patients with synchronous CRLM undergoing hepatectomy compared to a historical cohort, with 5-year survival rates of approximately 72% versus 44%.^34^ The authors attributed this improvement to increased use of neoadjuvant chemotherapy and adoption of liver-first or simultaneous liver-colon resection approaches.^34^ Our findings align with those of Bockhorn et al., who observed similar survival rates for patients with synchronous and metachronous CRLM.^36^ However, it is important to note that conflicting data exist, which report inferior overall survival for patients with synchronous hepatic metastases in both left- and right-sided CRC.^37^ The increased use of neoadjuvant chemotherapy, adoption of liver-first or simultaneous liver-colon resections and advancements in surgical techniques and perioperative care have most likely contributed to improvement of patient outcomes and survival when suffering from synchronous metastases. Our findings contribute to existing evidence that sequence of CRLM may be less critical than previously thought when considering surgical intervention.

Furthermore, our data indicate that iterative hepatic resection for recurrent CRLM does not negatively impact overall survival, consistent with current literature supporting this approach in selected patients.^17–19,38^ Wicherts and colleagues have demonstrated potential long-term survival benefits with iterative liver resections for relapsing CRLM, though careful patient selection is crucial.^17^ Evidence suggests that initial surgical procedure and liver tumor status minimally affect eligibility for subsequent hepatectomies, even following major or two-stage procedures.^18,19,39^ While recurrence rates remain high after two-stage hepatectomy, patients may still benefit from additional resections in terms of overall survival.^38^ Our analysis corroborates that type (anatomical vs. atypical) and extent (major vs. minor) of hepatic resection does not significantly influence overall survival outcomes. In our real-world surgical cohort, we observed a potential association between the initial surgical strategy and the timing of hepatic recurrence. This finding may be influenced by both clinical factors and underlying tumor biology. Given the limited number of patients in this cohort undergoing multiple hepatectomies and the heterogeneity in clinical variables, these observations should be interpreted with caution and considered hypothesis-generating rather than definitive.

Surgical resection of CRLU has demonstrated survival benefits in multiple studies.^15,40–43^ Prognostic factors influencing outcomes after pulmonary metastasectomy have been extensively investigated, with key factors including lymph node involvement, elevated pre-thoracotomy CEA levels, positive surgical margins, short disease-free interval, and number of pulmonary metastases.^9,15,42,44,45^ Multiple studies and systematic reviews consistently indicate improved survival for patients with solitary pulmonary lesions compared to those with multiple lesions, aligning with our findings.^15,44,46,47^ Our data further demonstrate a survival benefit for patients who have a limited number of metastases resected in the first pulmonary resection. Interestingly, while some publications identify larger lung metastases as a negative prognostic factor, our data suggest no correlation between size of pulmonary metastases and prognosis, warranting further investigation.^42,44,48,49^ The impact of pulmonary metastasectomy on OS with preexisting CRLM remains controversial, with conflicting results reported in the literature.^15,42,44,45,47,49,50^

Current data indicates that pulmonary metastasectomy significantly improves overall survival in patients with CRC metastases, regardless of prior hepatic resection history.^6,9,45,47,49^ Tanaka and colleagues demonstrate survival benefits for patients with simultaneous hepatic and pulmonary metastases undergoing metastasectomy compared to non-surgical treatment.^51^ In line, we have recently shown that patients suffering from CRC with hepatic and pulmonary metastases benefit from surgical resection of hepatic and pulmonary metastases in comparison to nonsurgically treated patients.^10^ Overall survival appears comparable between patients undergoing combined hepatic and pulmonary resection and those with isolated liver metastases resection.^23^

Our findings moreover suggest that iterative pulmonary metastasectomies are feasible and potentially beneficial for selected patients with recurrent CRLU, successfully ameliorating OS for solitary and recurrent CRLU. These data are in accordance with previous research demonstrating favorable outcomes through repeated surgical interventions in patients with resectable lung metastases and support considering a more aggressive surgical approach to recurrent pulmonary disease in carefully selected patients.^6,40,41,43,48^

While this study provides valuable insights into the management of metastatic CRC, it is important to acknowledge several limitations inherent to its retrospective, single-center design, underscoring the need for further investigations.

## Conclusions

Our data support the efficacy of aggressive surgical management for CRC metastases, including iterative hepatic and pulmonary resections. Patients with synchronous and metachronous metastases appear to benefit similarly from surgical intervention. Careful patient selection and interdisciplinary decision-making are crucial for optimal outcomes. A limited number of pulmonary metastases seems to emerge as prognostic factor, while the impact of metastasis size and prior hepatic resection remains controversial. Overall, these findings advocate for a more proactive surgical approach in selected patients with metastatic colorectal cancer, potentially improving long-term survival outcomes.

## Supplementary Information

Below is the link to the electronic supplementary material.

Supplementary file1 (PDF 589 kb)

## Data Availability

The dataset used and analyzed during the current study is available from the corresponding author on reasonable request.
