# Supplementary material for: Iterative Hepatic and Pulmonary Metastasectomy in Stage IV Colorectal Cancer: Impact on Survival and Surgical Outcomes
Source: Ann Surg Oncol. 2025 Sep 30;33(1):578–93. doi: 10.1245/s10434-025-18407-1 (PMC12689717; doi:10.1245/s10434-025-18407-1)
Supplement: Supplementary file 1 — Supplementary file1 (PDF 589 kb) [file 10434_2025_18407_MOESM1_ESM.pdf]

Supplemental Figures

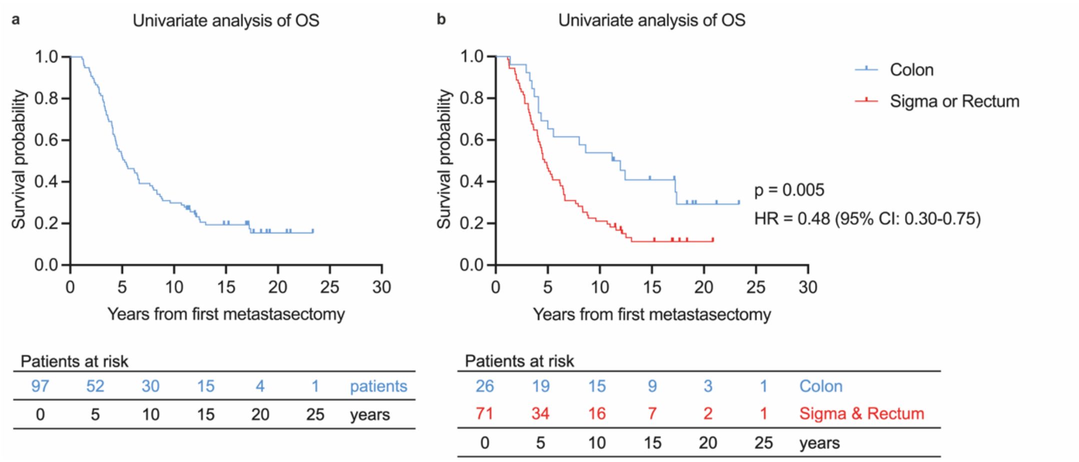

Supplemental Figure 1. (**a**) Kaplan-Meier curve of univariate overall survival in all patients. n = 97. Median survival estimate: 5.2 years; 95% CI: 3.8-6.7 years. (**b**) Kaplan-Meier curve of univariate overall survival in patients with colon or sigma and rectum carcinoma. n = 97. p = 0.005**.** HR (logrank) = 0.48 (95% CI: 0.30-0.75). "OS": overall survival.

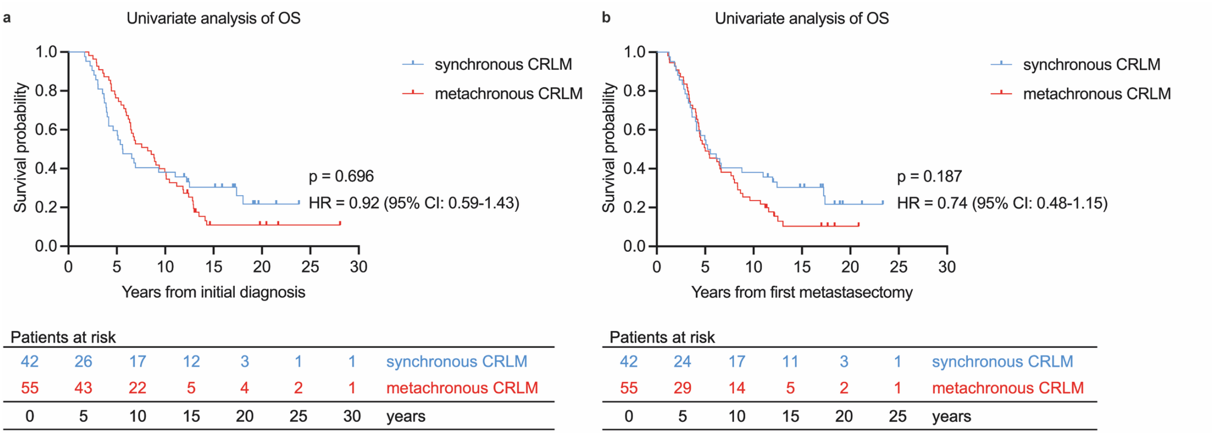

Supplemental Figure 2. **Sequence of hepatic metastasis.** Kaplan-Meier curve of univariate overall survival in patients with synchronous and metachronous CRLM. (**a**) Period from initial diagnosis to follow-up. n = 97. p = 0.696. HR (logrank): 0.92 (95% CI: 0.59-1.43). (**b**) Period from first metastasectomy to follow-up. n = 97. p = 0.187. HR (logrank) = 0.74 (95% CI: 0.48-1.15). "OS": overall survival.
